# Downregulation of leptin inhibits growth and induces apoptosis of lung cancer cells via the Notch and JAK/STAT3 signaling pathways

**DOI:** 10.1242/bio.017798

**Published:** 2016-05-16

**Authors:** Xian-Jie Zheng, Zhong-Xin Yang, Yan-Jun Dong, Guo-Yu Zhang, Ming-Fei Sun, Xiao-Kang An, Li-Hong Pan, Shuang-Lin Zhang

**Affiliations:** Department of Thoracic Surgery, The First Affiliated Hospital of Henan University, Kaifeng, Henan Province 475000, China

**Keywords:** Leptin, Non-small cell lung cancer, Proliferation, Apoptosis, Notch, JAK/STAT3

## Abstract

Previous studies have documented that leptin is involved in the pathogenesis of many human cancer types by regulation of numerous signal transduction pathways. The aim of this study was to investigate the biological roles of leptin and the mechanisms attributed to its action in non-small cell lung cancer (NSCLC) cell lines. The expression of leptin was measured by quantitative real-time PCR and western blot in seven NSCLC cell lines. Proliferation and apoptosis of NSCLC cells in response to leptin knockdown were determined by MTT assay and flow cytometry, respectively. The effect of leptin knockdown on the Notch and JAK/STAT3 signaling pathways was further examined by western blot. Leptin expression was significantly increased in NSCLC cell lines compared with normal human bronchial epithelial cell HBE. Leptin knockdown inhibited cell proliferation and induced apoptosis in NSCLC cell lines through inactivation of the Notch and JAK/STAT3 signaling pathways. Furthermore, gene silencing of Notch signaling with Notch-1 siRNA or inhibition of JAK/STAT3 signaling by JSI-124, an inhibitor of STAT3, resulted in proliferation inhibition and apoptosis induction in NSCLC A549 cells. Our findings suggested that leptin knockdown could become a new approach for the prevention of lung cancer progression, which is likely to be mediated at least partially by inactivation of the Notch and JAK/STAT3 signaling pathways.

## Abstract (precis)

**Summary:** Leptin knockdown could become a new approach for the prevention of lung cancer progression, which we have shown is likely to be mediated by inactivation of the Notch and JAK/STAT3 signaling pathways.

## INTRODUCTION

Lung cancer is the leading cause of cancer deaths worldwide, and has high incidence rates (Ferlay et al., 2010). Non-small cell lung cancer (NSCLC) accounts for approximately 85% of all lung cancers and its 5-year survival rate hardly reaches 15% (Liu et al., 2014). Traditional treatments including surgery, radiotherapy and chemotherapy are still relatively ineffective for patients suffering lung cancer (Brunelli et al., 2009; Kennedy, 2005). As understanding of the molecular basis of tumorigenesis improves, use of molecular targets may contribute to novel treatment approaches for lung cancer.

Leptin, a 16-kDa nonglycosylated protein encoded by the Ob gene and mainly secreted by adipose tissue (Friedman and Halaas, 1998), is a protein hormone with relevant roles in cell survival, differentiation, inflammation and angiogenesis which indicate its potential activities in cancer development and progression (Sierra-Honigmann et al., 1998; Somasundar et al., 2004). It has been reported that leptin expression is highly correlated with expression levels of human telomerase reverse transcriptase (hTERT) and could affect cancer progression and invasion in hepatocellular carcinoma (HCC) (Stefanou et al., 2010). Similarly, expression levels of leptin correlated positively with the grades of cervical carcinoma as well as expression of c-myc and bcl-2, indicating the regulatory roles of leptin in cell proliferation and apoptosis in cervical cancer cells (Yuan et al., 2013). In lung cancer, previous studies have reported that elevated serum leptin concentration in NSCLC patients may be involved in the development of non small-cell lung carcinogenesis independent of central obesity (Terzidis et al., 2009).

Leptin exerts its various physiological activities through the six isoforms of Ob-R, a single membrane-spanning receptor, which belongs to the superfamily of cytokine receptor I (Margetic et al., 2002). Leptin signaling pathway is thought to be transmitted through activation of Ob-R that induces several canonical and non-canonical signaling pathways, including JAK/STAT, MAPK, PKC, JNK and PI3K/AKT, to exert its biological functions in food intake, energy balance, and adiposity as well as tumorigenesis (Choi et al., 2005; Saxena et al., 2007; Yan et al., 2012). Binding of leptin to Ob-R leads to tyrosine phosphorylation of JAK1 and JAK2, as well as the homodimerization of Ob-R and the phosphorylation of downstream transcription factors STATs (Ahima and Osei, 2004). Prior investigation showed that leptin promotes hepatocellular carcinoma growth, invasiveness, and migration through concomitant activation of the JAK/STAT, PI3K/AKT and ERK signaling, which implicates the JAK/STAT pathway as a critical mediator of leptin action (Saxena et al., 2007).

The Notch family consists of four receptors (Notch 1–4) and has five ligands named Jagged 1 (JAG1), JAG2, Delta-like 1 (DLL1), DLL3 and DLL4 (Shawber et al., 2003). Notch signaling has been proposed to influence many different types of cell-fate decision from proliferation, differentiation and apoptosis to cancer cell invasion and metastasis (Cheng et al., 2014). Although the exact role of Notch signaling pathway in human lung cancer is still not fully clarified, fetal lung developmental studies suggest the critical role of Notch signaling in the development of airway epithelial cells (Watkins et al., 2003). According to current research, pharmacologic inhibition of the Notch pathway is regarded as a potentially experimental approach for the treatment of non-small cell lung cancers (Liu et al., 2015). Previous studies showed that leptin activated Notch signaling, which could be involved in the proliferation and migration of breast cancer cells and poor prognosis of breast cancer (Battle et al., 2014).

In the present study, we investigated for the first time the effect of targeted deletion of leptin on NSCLC cells. We also attempted to explore the molecular pathways that mediate this effect by measuring the activation of the Notch and JAK/STAT3 signaling pathways. In addition, the Notch and JAK/STAT3 signaling pathways were inhibited by Notch-1 siRNA and JSI-124, respectively, to determine the roles of these pathways in lung cancer cells.

## RESULTS

### Leptin expression was downregulated in NSCLC cell lines

**Fig. 1.**
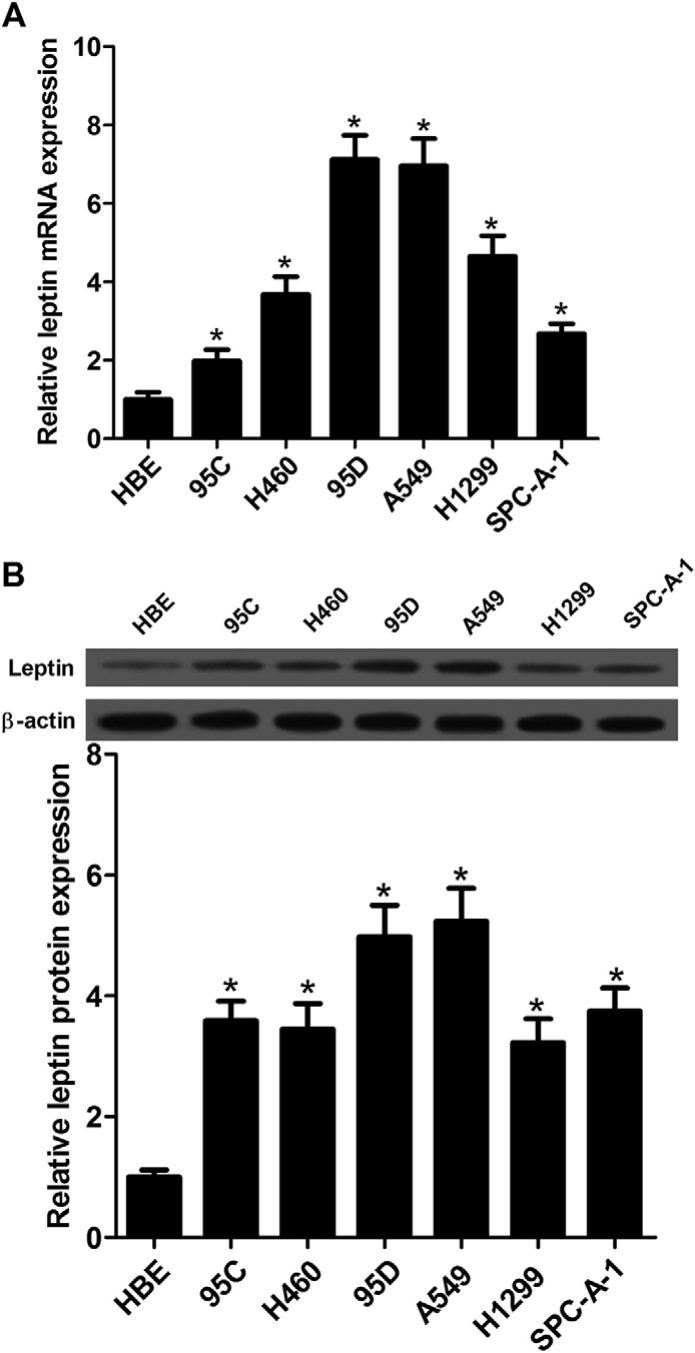
**Detection of leptin expression in NSCLC cell lines.** (A) Levels of leptin expression in HBE, 95C, H460, 95D, A549, H1299 and SPC-A-1 cells were determined by qRT-PCR. (B) Western blot analysis was employed to detect the protein expression levels of leptin in NSCLC cell lines. Data expressed as mean± s.e.m.; *P<0.05 compared to HBE cells.

To measure the biological roles of leptin in NSCLC cells, we first determined the expression of leptin in NSCLC cell lines including 95C, H460, 95D, A549, H1299, SPC-A-1 and in normal human bronchial epithelial cell HBE by qRT-PCR and western blot. The results showed that the mRNA expression of leptin was significantly increased in six NSCLC cell lines compared with HBE (Fig. 1A). Similarly, western blot analysis showed that compared with HBE cells protein expression levels of leptin was significantly elevated in NSCLC cell lines (Fig. 1B).

### Silencing of leptin reduced cell proliferation in NSCLC cells

**Fig. 2.**
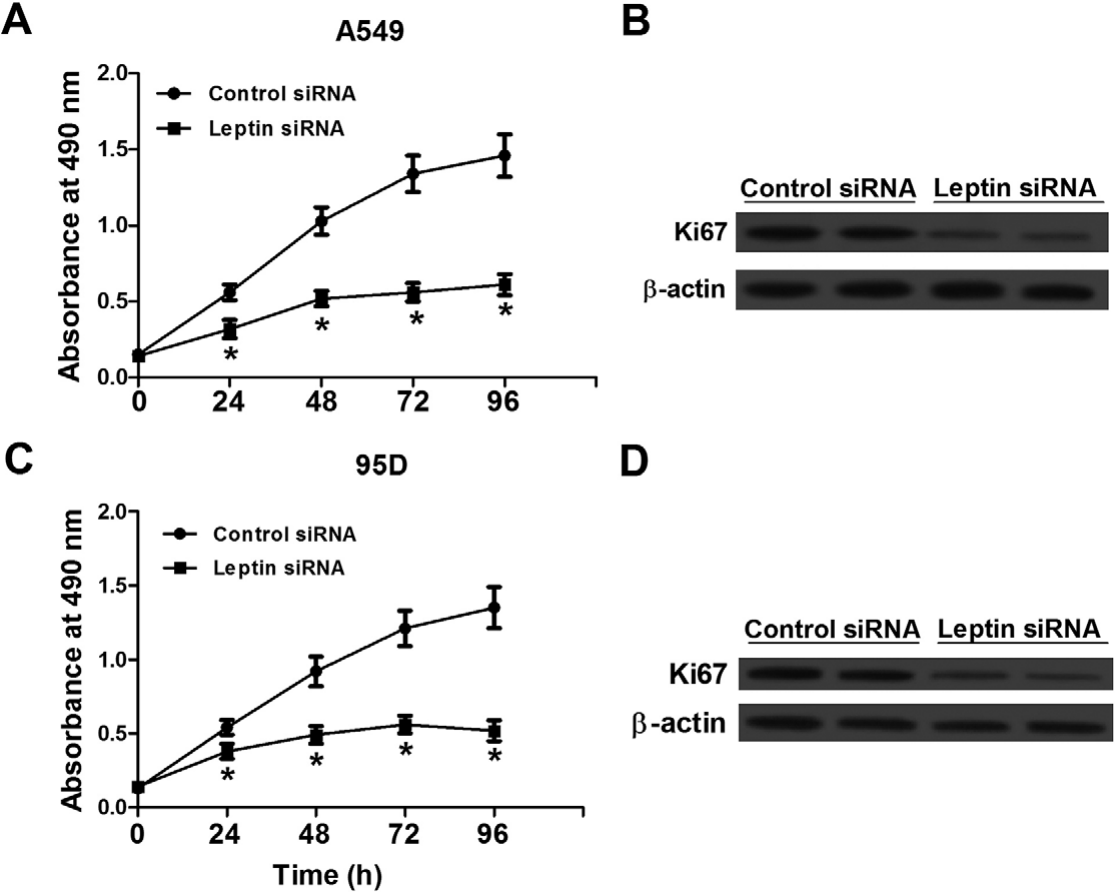
**Silencing of leptin by leptin-targeted siRNA reduced cell proliferation in NSCLC A549 and 95D cells.** (A,C) Growth curves calculated for A549 and 95D cells treated with leptin siRNA and control siRNA. (B,D) Ki67 expression A549 and 95D cells treated with siRNA transfection. Western blot analysis showed that Ki67 expression in cells treated with leptin siRNA was significantly lower than that of cells treated with control siRNA. Data expressed as mean± s.e.m.; *P<0.05 compared to control siRNA-treated cells.

To measure the effect of leptin knockdown on NSCLC cell viability, MTT assay was used to determine growth activity of A549 and 95D cells transfected with leptin siRNA or control siRNA (Fig. 2A,C). The results showed that all of the two cell lines transfected with leptin siRNA displayed lower proliferation rates compared with cells with control siRNA. Moreover, in accordance with the cell viability, the protein expression of Ki67 in leptin siRNA-transfected cells also decreased significantly compared with control siRNA-transfected cells (Fig. 2B,D).

### Silencing of leptin induced apoptosis in NSCLC cells

**Fig. 3.**
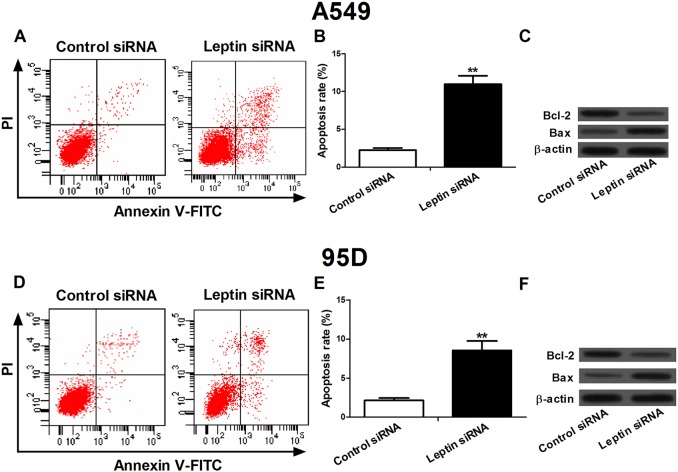
**Silencing of leptin by leptin-targeted siRNA induced apoptosis in NSCLC A549 and 95D cells.** (A,D) Flow cytometry analysis was used to detect the apoptosis in A549 and 95D cells after siRNA transfection. (B,E) The apoptosis rate in A549 and 95D cells. Cells transfected with leptin siRNA presented more apoptosis compared with the cells transfected with control siRNA. (C,F) Expression levels of Bcl-2 and Bax in A549 and 95D cells after siRNA transfection. Leptin RNAi significantly decreased Bcl-2 expression and increased Bax expression in both the NSCLC cell lines. Data expressed as mean± s.e.m.; **P<0.01 compared to control siRNA-treated cells.

To investigate the effect of leptin knockdown on apoptosis, the flow cytometry was used to detect the apoptosis rate in A549 and 95D cells transfected with leptin siRNA or control siRNA (Fig. 3A,B,D,E). After siRNA transfection for 48 h, the apoptosis rates in A549 and 95D cells transfected with leptin siRNA were significantly increased compared with control siRNA-transfected cells. In addition, we measured the expression of Bcl-2 and Bax using western blot (Fig. 3C,F). The results showed that compared with cells receiving control siRNA, the protein expression of Bax was increased significantly, whereas the expression of Bcl-2 was significantly downregulated in both A549 and 95D cells treated with leptin siRNA.

### Effect of leptin knockdown on the Notch and JAK/STAT3 signaling pathways

**Fig. 4.**
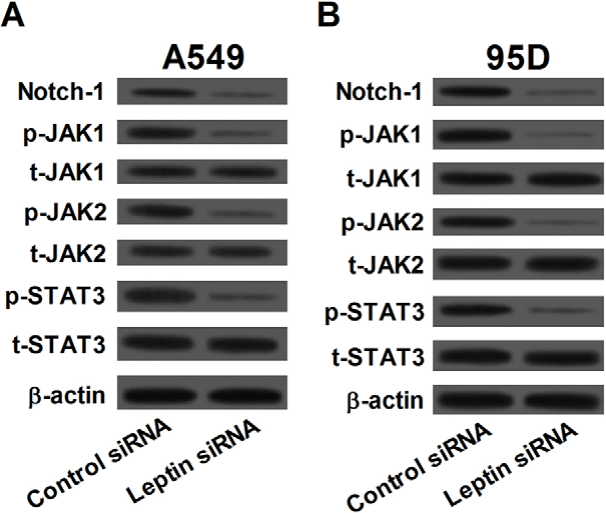
**Silencing of leptin inactivated the Notch and JAK/STAT3 signaling pathways.** In both A549 (A) and 95D (B) cells, knockdown of leptin led to decreased levels of Notch-1, phosphorylated-JAK1 (p-JAK1), p-JAK2 and p-STAT3 but not total-JAK1 (t-JAK1), t-JAK2, and t-STAT3.

Expression levels of Notch-1, phosphorylated-JAK1 (p-JAK1), p-JAK2, p-STAT3, total-JAK1 (t-JAK1), t-JAK2, and t-STAT3 were measured by western blot to evaluate the effect of leptin knockdown on the Notch and JAK/STAT3 signaling pathways (Fig. 4A,B). In both A549 and 95D cells, leptin siRNA treatment for 48 h significantly downregulated the expression of Notch-1, p-JAK1, p-JAK2, and p-STAT3 but not t-JAK1, t-JAK2, and t- STAT3 compared with control siRNA treatment. The results indicated that knockdown of leptin led to inactivation of both the Notch and JAK/STAT3 signaling pathways in NSCLC cells.

### Notch-1 siRNA inhibited cell proliferation and induced apoptosis in A549 cells

**Fig. 5.**
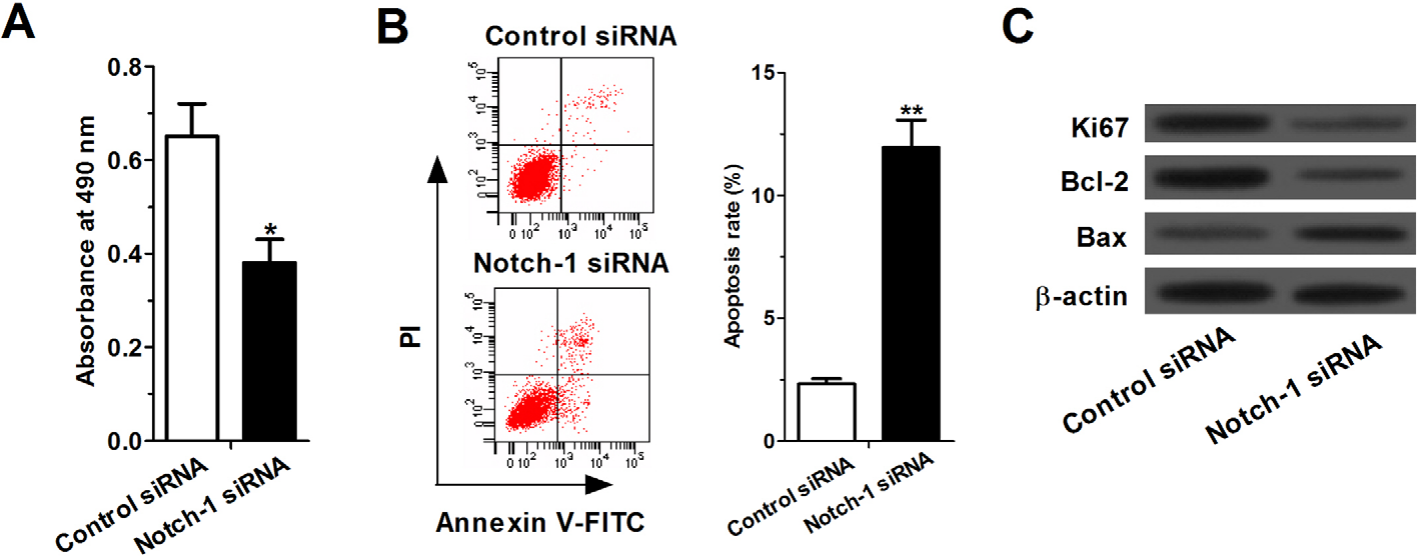
**Notch-1 siRNA inhibited cell proliferation and induced apoptosis in A549 cells.** (A) MTT assay showed that the growth rate of the Notch-1 siRNA-treated cells was significantly lower than the control siRNA-treated cells after transfection for 48 h (B) Flow cytometry analysis revealed that Notch-1 siRNA significantly induced apoptosis in A549 cells. (C) The expression levels of Ki67 and Bcl-2 were markedly downregulated and Bax expression was upregulated in Notch-1 siRNA-treated cells compared with control siRNA-treated cells. Data expressed as mean± s.e.m.; *P<0.05 and **P<0.01 compared to control siRNA-treated cells.

Our study suggested that leptin knockdown inactivated Notch signaling, which played a role in proliferation and apoptosis in NSCLC cells. We sought to determine whether the inhibition of Notch signaling by Notch-1 siRNA suppressed cell proliferation and induced apoptosis in A549 cells. MTT assay showed that the growth rate of the Notch-1 siRNA-treated cells was significantly lower than the control siRNA-treated cells after transfection for 48 h (*P*<0.05) (Fig. 5A). As shown in Fig. 5B, flow cytometry analysis revealed that Notch-1 siRNA significantly induced apoptosis in A549 cells (*P*<0.01). Additionally, the expression levels of Ki67 and Bcl-2 were markedly downregulated and Bax expression was upregulated in Notch-1 siRNA-treated cells compared with control siRNA-treated cells (Fig. 5C).

### JSI-124 suppressed cell proliferation and induced apoptosis in A549 cells

**Fig. 6.**
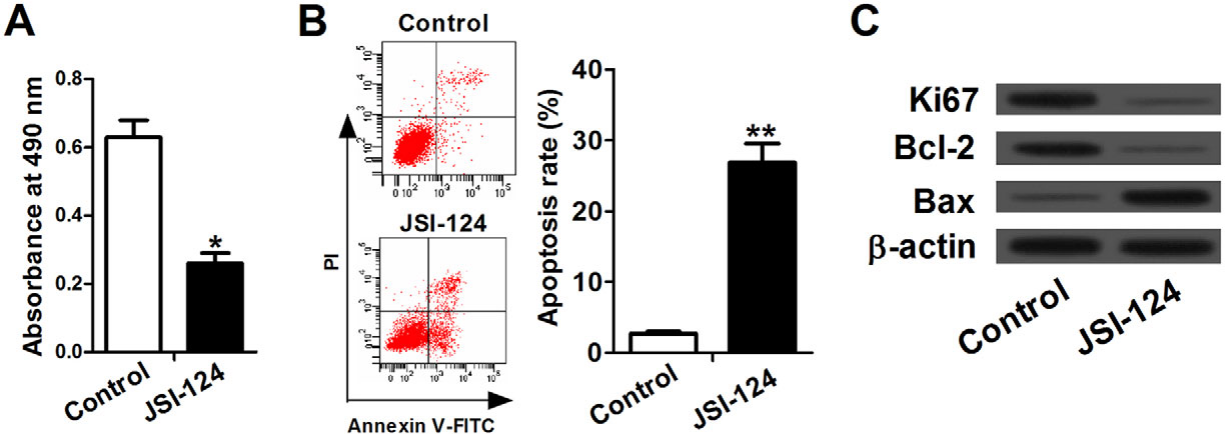
**Inactivation of JAK/STAT3 signaling pathway suppressed cell viability and induced apoptosis in A549 cells.** Cells were treated with 1 μM JAK/STAT3signaling pathway inhibitor, JSI-124 for 12 h. (A) Cell proliferation was measured by MTT assay. JSI-124 treatment significantly inhibited the growth of A549 cells compared with control group. (B) Flow cytometry analysis was used to detect the apoptosis in A549 after JSI-124 treatment. (C) The expression levels of Ki67 and Bcl-2 were markedly downregulated and Bax expression was upregulated in JSI-124-treated cells compared with control group. Data expressed as mean±s.e.m.; *P<0.05 and **P<0.01 compared to control group.

A549 cells were treated with 1 μM JAK/STAT3 signaling pathway inhibitor, JSI-124, for 12 h to further understand JAK/STAT3 signaling in leptin knockdown-induced cell proliferation inhibition and apoptosis induction in NSCLC cells. As shown in Fig. 6A, JSI-124 treatment significantly inhibited the growth of A549 cells compared with control group (*P*<0.05). Results from flow cytometry analysis showed that JSI-124 treatment significantly increased apoptosis in A549 cells compared with control group (*P*<0.01) (Fig. 6B). Furthermore, the expression levels of Ki67 and Bcl-2 were markedly downregulated and Bax expression was upregulated in JSI-124-treated cells compared with control group (Fig. 6C).

## DISCUSSION

In the current study, we first measured the prevalence of the expression of leptin in NSCLC cell lines including 95C, H460, 95D, A549, H1299 and SPC-A-1 by qRT-PCR and western blot. Results from both mRNA and protein expression analysis showed that leptin expression was significantly increased in six NSCLC cell lines compared with normal human bronchial epithelial cell HBE. We also found that deletion of leptin could inhibit cell proliferation and induce apoptosis in NSCLC A549 and 95D cells, as determined by MTT assay and flow cytometry analysis, respectively. The Notch and JAK/STAT3 signaling pathways were investigated to clarify the mechanism by which silencing of leptin inhibited cell proliferation induced apoptosis in NSCLC cells.

Leptin has been reported to promote proliferation in various types of cancer cells. Leptin promoted cell proliferation and homotypic tumor cell adhesion through increased expression of E-cadherin and cyclin D1 *in vivo* in breast cancer xenograft and *in vitro* in breast cancer cell lines (Mauro et al., 2007). Leptin also positively regulated endometrial cancer growth via JAK/STAT and AKT pathways (Sharma et al., 2006). Previous studies have shown that leptin stimulated the proliferation of hepatocellular carcinoma HepG2 cells in a time- and dose-dependent manner, and knockdown of leptin resulted in notable reduction in proliferation rate (Stefanou et al., 2010). In contrast, reduced leptin expression was reported to reverse cell proliferation and induce apoptosis in multiple cancer cells (Cha et al., 2012; Yuan et al., 2013). Here, after NSCLC A549 and 95D cells were treated with siRNA, compared with control siRNA-treated cells proliferation rates were significantly decreased in cells treated with leptin siRNA. Furthermore, leptin siRNA inhibited the expression levels of proliferation marker Ki-67. These data were similar to the findings of previous studies in cervical cancer cells (Yuan et al., 2013).

Leptin represented anti-apoptotic activities in many human cancer cells including Barrett's esophageal adenocarcinoma cells, colon cancer cells, and breast cancer cells (Jardé et al., 2011; Ogunwobi et al., 2006; Ogunwobi and Beales, 2007; Rouet-Benzineb et al., 2004). In Barrett's esophageal adenocarcinoma cells, leptin has been reported to stimulate cell proliferation and impede apoptosis via a complex cascade of reactions (Ogunwobi et al., 2006). In human colon cancer cells, leptin could also promote proliferation and inhibit apoptosis via activation of JNK mitogen activated protein kinase, JAK2 and PI3 kinase/Akt (Ogunwobi and Beales, 2007). Previous studies showed that leptin reversed sodium butyrate-induced apoptosis in human colon cancer HT-29 cells through MAP kinase and NF-κB pathways (Rouet-Benzineb et al., 2004). In our study, after NSCLC A549 and 95D cells were treated with leptin siRNA, flow cytometry analysis showed that the apoptosis rates were significantly increased. Taken together, the results indicated a molecular link between leptin knockdown and viability as well as apoptosis in NSCLC cells, providing supporting evidence that leptin represents a target for lung cancer therapy.

Leptin significantly elevated the expression levels of Notch1-4, Notch target genes, Hey2 and increased survival in breast cancer cells; in addition, leptin is an inducer of Notch signaling through regulating Notch1-4 expression and/or activation (Guo and Gonzalez-Perez, 2011). More recent studies showed that leptin induced expressions of Notch1, 3, 4 in breast cancer cells, and inhibition of leptin signaling, led to decreased protein expression levels of NICD1, NICD4, Notch3, JAG1 and survivin as well as reduced mRNA levels of Notch receptors, ligands and targets (Battle et al., 2014). Here, after NSCLC cells were treated with siRNA against leptin Notch-1 was significantly downregulated, and targeted deletion of Notch-1 suppressed cell proliferation and induced apoptosis in A549 cells. These data suggested that Notch signaling might be involved in the leptin knockdown-induced cell death and apoptosis.

Previous studies showed that leptin promoted viability and metastasis of renal cell carcinoma cells via activating the ERK1/2 and JAK/STAT3 signaling which could be partially abolished by ERK phosphorylation inhibitor U0126 and STAT3 phosphorylation inhibitor AG490, respectively (Li et al., 2008). Other studies indicated that concomitant activation of the JAK/STAT, PI3K/AKT and ERK signaling played crucial roles in leptin-mediated invasion and metastasis of hepatocellular carcinoma cells (Saxena et al., 2007). According to the experimental evidence that leptin, JAK/STAT3, and Notch are intimate partners in crime with regard to tumorigenesis, we hypothesize that targeting inactivation of these pathways by leptin knockdown may be a novel treatment approach for lung cancer. Our findings indicate that leptin siRNA transfection could decrease the p-JAK1, p-JAK2, and p-STAT3 expression in NSCLC cells, and inhibition of JAK/STAT3 signaling could inhibit cell growth and induce apoptosis in NSCLC A549 cells, indicating the involvement of a JAK/STAT3 pathway in leptin silence-induced proliferation inhibition and apoptosis induction.

In summary, our results strongly suggested that for the first time, the role of leptin deletion could be a potential antitumor approach toward the treatment of lung cancer. Moreover, our experimental evidence provides mechanistic information indicating that leptin knockdown-induced cell proliferation inhibition and apoptosis induction are likely to be mediated, at least partially, by inactivation of the Notch and JAK/STAT3 signaling pathways.

## MATERIALS AND METHODS

### Cell culture

The NSCLC cell line A549 and human bronchial epithelial cell HBE were obtained from American Type Culture Collection (ATCC, Manassas, VA, USA). The NSCLC cell lines 95D, 95C H460, SPC-A-1 and H1299 were purchased from the Cell Bank of Type Culture Collection of Chinese Academy of Sciences (Shanghai, China). HBE cells were grown in DMEM medium (Invitrogen, Gaithersburg, MD, USA) and all the NSCLC cell lines were cultured in RPMI 1640 medium (Invitrogen) supplemented with 10% fetal bovine serum, penicillin (100 U/ml) and streptomycin (100 µg/ml), in an incubator with 5% CO_2_ at 37°C.

### RNA interference

The A549 and 95D cells were transfected with leptin siRNA or scramble control siRNA (Dharmacon, Lafayette, CO, USA) using Lipofectamine 2000 reagent as suggested by the manufacturer (Invitrogen). In addition, the A549 cells were transfected with Notch-1 siRNA and siRNA control (Santa Cruz Biotechnology, Santa Cruz, CA, USA) using Lipofectamine 2000 reagent (Invitrogen). All the cells were harvested 48 h after transfection.

### Cell proliferation assay

Forty-eight hours after transfection, cells were seeded at a density of 5×10^3^ cells/well in 96-well culture plates for 24, 48, 72, and 96 h. Then, cells were incubated with 0.5 mg/ml MTT reagent (Sigma, St. Louis, MO, USA) for 4 h at 37°C. Four hours later, the medium was replaced with 100 μl/well of DMSO (Sigma) to dissolve the remaining formazancrystals and the absorbance of each well at 490 nm was recorded.

### Flow cytometry analysis

Forty-eight hours after transfection, the exponentially growing cells were trypsinized and washed in cold PBS. After washes, cells (1×10^6^/well) were resuspended in binding buffer. Annexin V-FITC and propidium iodide (PI) were added in each well, according to the Annexin V-FITC apoptosis Detection Kit (BD Pharmingen, San Diego, CA, USA). The apoptosis of each sample was monitored by fluorescence activated cell sorter (FACS).

### Quantitative real-time PCR

Total RNA for quantitative real-time PCR (qRT-PCR) was extracted using TRIzol reagent (Invitrogen). Reverse transcription reactions were performed using the TaqMan Reverse Transcription Reagents (Applied Biosystems, Foster City, CA, USA). For analysis of mRNA expression, real-time PCR was carried out by SYBR Green Reagents (Applied Biosystems) according to the manual. Relative expression of each sample was normalized to β-actin mRNA and calculated by the ΔΔCt method. The primers were synthesized by Shanghai Sangon Biological Engineering Technology & Services (Shanghai, China).

### Western blot analysis

Total protein was extracted from cells using RIPA kit (Pierce Biotechnology, Rockford, IL, USA) followed by the BCA protein assay to determine protein concentration. Equal amounts of protein were electrophoresed on a polyacrylamide gel and transferred onto polyvinylidene fluoride (PVDF) membrane (Millipore, Bedford, MA, USA). The membrane was incubated with anti-leptin, anti-Notch-1 (sc-373891), anti-total (t)-JAK1, anti-t-JAK2, JAK1 phospho (p)-Tyr1022/1023, JAK2 p-Tyr1007/1008, anti-t-STAT3, STAT3 p-Tyr705, and anti-β-actin, all of which were purchased from Santa Cruz Biotechnology. After washes, the membrane was incubated with horseradish peroxidase (HRP)-conjugated secondary antibodies (Cell Signaling, Beverly, MA, USA). Bound antibody was detected with enhanced chemiluminescence (ECL) kit (Amersham Pharmacia Biotech, Piscataway, NJ, USA). β-actin served as an internal control.

### Statistical analysis

The results were expressed as mean±standard errors (s.e.m.). Differences between two groups were analyzed using the Student's *t*-test. Comparisons of more than two data sets were performed using one-way ANOVA. *P*-values <0.05 were considered to be statistically significant.
